# If Hunters End Up in the Emergency Room: A Retrospective Analysis of Hunting Injuries in a Swiss Emergency Department

**DOI:** 10.1155/2015/284908

**Published:** 2015-03-05

**Authors:** Valentina Bestetti, Emma E. Fisher, David S. Srivastava, Meret E. Ricklin, Aristomenis K. Exadaktylos

**Affiliations:** Department of Emergency Medicine, University Hospital and University of Bern, Freiburgstrasse 16c, 3010 Bern, Switzerland

## Abstract

*Aim*. to characterize the mechanisms, patterns, and outcomes of nonfatal hunting-related injuries in patients presenting to Bern University Hospital, Switzerland, and compare these to reports of hunting injuries worldwide. *Methods*. patients presenting with hunting-related injuries to the Emergency Department at Bern University hospital from 2000 to 2014 were identified by retrospectively searching the department database using the keyword “hunt.” Each case was analyzed in terms of the patient age and gender, the mechanism and pattern of injury, and management and patient follow-up. *Results*. 19 patients were identified. 16 were male with a mean age of 50 years (range: 16–74). Mechanisms of injury included firearm-related injuries, falls, and knife wounds. The most common patterns of injury were head injuries (7), followed by injuries to the upper (5) or lower limb (5) and trunk (2). Over half of the patients were admitted, and nine required emergency surgery. *Conclusion*. Nonfatal hunting accidents in Bern, Switzerland, are largely caused by firearms and falls and tend to occur in male hunters with a mean age of 50 years. The most common patterns of injury are orthopedic and head injuries, often requiring surgery. These findings are consistent with international studies of nonfatal hunting accidents.

## 1. Introduction

Hunting is a popular hobby in Switzerland, involving about 30,000 people each year [1]. Hunting injuries, especially those that have fatal consequences, attract a great deal of media attention. However, the literature on hunting injuries, particularly those that are nonfatal, is disappointingly scarce. This is most likely due to underreporting of nonfatal hunting accidents and a lack of national databases for recording these incidents in regions where hunting is popular, including Switzerland, Scandinavia, and Germany. In Switzerland, it is only compulsory to report fatal hunting accidents to the Advisory Centre for Accident Prevention. In contrast, the National Electronic Injury Surveillance System (NEISS) and the International Hunter Education Association (IHEA) in the USA collect data about both fatal and nonfatal hunting accidents across the country. The USA has therefore provided much of the existing data on the causes, mechanisms, and outcomes of nonfatal hunting accidents as well as the characteristics of the hunters involved.

Hunting is an even more popular recreational activity in the USA than in Switzerland; in 2014 over 16 million Americans obtained a hunter's license, which equates to about 5% of the total population [2]. According to the NEISS, which collects data from one hundred emergency departments across the USA, over 7,000 Americans were involved in hunting accidents due to firearms or tree-stand falls in 2013 [3]. Most of these accidents were nonfatal. However, the IHEA, who also have a key role in gathering data about hunting-related incidents, estimates that 10% of the 1000 hunting-related firearm injuries recorded annually in USA are fatal [4].

In terms of hunter demographics, studies of hunting-related accidents from Denmark, Germany, and the USA have discovered that those involved tend to be males with hunting experience and with a mean age of 40–50 years old [5–8]. Younger hunters appear to be involved in fewer accidents, despite evidence that they engage in more risky hunting behaviors, such as use of camouflage [5, 9]. There is also evidence from studies of Danish and Swedish hunters that people involved in accidents tend to be those that hunt most frequently [5, 9].

Regarding hunting accidents themselves, most occur during the hunt rather than at home or in the car before or after the hunt [6, 9]. The majority are caused by firearms or falls from hides [10]. Much of the literature to date has focused on hunting accidents caused by the former. The firearm injury surveillance study found that there were 1.8 million presentations of firearm-related injuries across emergency departments in the USA from 1993 to 2008 and just under 2% of these were caused by hunting accidents [11]. Reasons for firearm-related hunting accidents include not seeing the victim, not using the weapon appropriately, swinging the weapon whilst shooting, and, less commonly, mistaking the victim for game [5, 6].

Research has shown that the pattern of injury depends on the mechanism of the hunting accident. A study of deer hunting-related injuries in Wisconsin found that injuries from firearms were evenly split between orthopedics and general surgery, whilst 90% of tree-stand related injuries were orthopedic in nature [7]. Common orthopedic injuries due to falls from tree stands include spinal, pelvis, and extremity fractures [10]. Tree-stand falls also carry a substantial risk of high impact head injury and neurological deficit [12].

With respect to the prevention of hunting accidents, a study of the behavior and attitudes of Swedish hunters found that most believe safer weapon handling and better hunt planning can prevent hunting accidents [9]. In accordance with these findings, efforts to minimize hunting-related accidents in most countries have focused on increasing the visibility of hunters by encouraging them to don “hunters' orange” and educating hunters in gun safety [10]. However, hunting injuries appear to be on the increase in the USA despite these measures, and recently attention has turned to the safety issues surrounding the use of tree stands for large game hunting [10, 12].

The aim of this study is to analyze the causes, patterns, and management and outcomes of nonfatal hunting-related injuries in a series of patients presenting to Bern University hospital, Switzerland, and to compare these to reports of hunting injuries worldwide. To our knowledge, this is the first time that data on nonfatal hunting accidents in Switzerland has been analyzed in this way.

## 2. Materials and Methods

The Emergency Department of Bern University Hospital provides emergency care for a population of 1.8 million inhabitants and treats more than 35,000 adults each year.

Our study covers an observational period of 14 years, from November 2000 to November 2014, and includes adult patients treated in the Emergency Department of Bern University Hospital for hunting-related injuries.

The emergency department database, starting in 2000, of health-related patient data allows authorized healthcare professionals to access clinical reports, radiographs, laboratory results, and other relevant medical documents. During the observational period, two database systems were implemented: Qualicare (Qualidoc AG, Trimbach, Switzerland) was used from November 2000 to July 2012 and E.Care (E.care bvba, Turnhout, Belgium) from July 2012 to November 2014.

The database search was performed by the emergency department's database manager using the keyword “hunt.” The search also included possible misspellings of this word to optimise the search results.

The data were then reviewed by the first author to check the relevance of the search results to the study. Cases that were judged relevant by the first author were classified by gender, age, mechanism and pattern of injury, management, and the need for follow-up at one month and one year.

## 3. Results

### 3.1. Hunter Demographics

From 2000 to 2014, 19 patients presented to Bern University Emergency Department with hunting-related injuries. 16 of these patients were male and the mean age was 50 years (range: 16–74 years).

### 3.2. Mechanisms of Injury

Most injuries (*n* = 18; 95%) were self-inflicted; only one person in this study was shot by a third party. Four patients were admitted with injuries from firearm projectiles and three with injuries from firearms backfiring. Four patients sustained penetrating knife wounds and six patients were admitted with injuries related to falls whilst hunting, be this from an elevated hunting stand or walking in the wilderness. One patient was admitted with sciatica and another with footwear-related pressure sores. See Table 1.

### 3.3. Patterns of Injury

The patterns of hunting-related injuries were also analysed. Seven patients suffered head injuries, five sustained injuries to the upper limb, and another five patients injured the lower limb. Two patients sustained injuries to the trunk. See Table 2.

### 3.4. Management of Hunting-Related Injuries

Over half of the patients (*n* = 11) with hunting-related injuries were admitted and the remaining eight were treated in the emergency department and discharged without any further complications. Eight of the eleven inpatients required emergency surgery: four required emergency orthopaedic surgeries for soft tissue injuries or in one case, subluxation of the hip; three required urgent hand surgery for soft tissue injuries and one patient underwent neurosurgery for a subdural haematoma. The three remaining patients that were admitted did not require surgery and were managed conservatively by various specialties (cardiology, rheumatology, and ENT).

### 3.5. Follow-Up

Five of the patients that were admitted were discharged with no further follow-up and did not return to hospital within the first year following their accident. Three of the inpatients were discharged with outpatient follow-up scheduled within 30 days of discharge. One patient required a plaster cast for two months and was then successfully discharged from outpatient care. Another patient required a longer follow-up period of three months, during which he had five outpatient appointments. He was then discharged from outpatient care without further complications. The patient that underwent neurosurgery was readmitted 20 days after discharge due to relapse of the subdural hematoma. No further information was available on the emergency department database about his follow-up since his second admission.

## 4. Discussion

Hunting continues to be a popular pastime for many people in Switzerland. Hunting accidents are inevitable and those that are fatal must be reported to the Swiss Advisory Centre for Accident Prevention. However there is no national registry of nonfatal hunting accidents in Switzerland in contrast to the USA where two organizations systematically document fatal and nonfatal hunting accidents nationwide. Until now, the mechanisms, patterns of injury, and outcomes of nonfatal hunting accidents in Switzerland have not been investigated. To our knowledge, this is the first Swiss study to examine hunting-related injuries, with respect to patient characteristics, mechanisms and patterns of injury, management, and patient outcomes.

The results of this study are largely consistent with the limited existing international data on nonfatal hunting accidents. We found that most nonfatal hunting accidents involved men with a mean age of 50 years, which is similar to the results of studies from Denmark, Germany, and the USA [5–8].

In addition, the most commonly involved weapon was a shotgun, followed by knives. This finding supports the results of Therbo and Van der Osten who found that 88% of hunting accidents in a group of Danish hunters were caused by shotguns and only 6% by knives [5]. Another finding from this study was that most injuries were self-inflicted rather than caused by another person: this supports the finding that over half of hunting-related accidents in central Ohio are self-inflicted [8]. In contrast, studies of German and Danish hunters have found that the majority of fire-arm-related hunting injuries involve a third party [5, 6]. One possible explanation for these mixed results could be differences in the teaching and content of firearm training and hunting examinations in different countries.

Our results support recent evidence from the USA that falls, as opposed to direct injury from firearm projectiles, are the principal cause of nonfatal hunting-related injuries [10, 12]. In this study, over almost a third of hunting-related injuries were associated with falls but not all from elevated tree stands; some injuries were sustained from falls whilst hunting in difficult terrain. In the USA, falls from tree stands may account for up to two-thirds of all hunting-related injuries and 8% result in permanent neurological injury or death [7, 8]. Common causes for falls from tree stands include poor tree stand construction, loss of balance, and falling asleep in the hide [12].

The results of this study also reveal that the most common patterns of injury from nonfatal hunting accidents are injuries to the head and extremities; however, in this study, no fractures were recorded. This finding supports other studies that have shown a high incidence of head injuries in hunters that fall from tree stands. About 60% of the patients in this study required operative intervention, which is 20% less than what Crockett et al. found in their study of hunting-related injuries in Ohio [8]. The most frequently involved specialty in the management of our series of hunting-related accidents was orthopedics, which is consistent with findings of Halanski and Corden [7].

Analysis of patient follow-up demonstrates that most patients that are admitted after hunting-related accidents can be discharged after initial management of their injuries with no further or very minimal follow-up as outpatients. Similarly, in a study of neurological injuries from hunting-related tree stand falls in New York, 72% of patients were discharged without follow-up and a further 11% with outpatient follow-up [12]. Two patients in our study required an extended period of follow-up before being discharged successfully, and only one patient in this case series required readmission.

Whilst our study may offer a unique insight into nonfatal hunting injuries in Switzerland, it is not without its limitations. Firstly, this study only evaluated nonfatal hunting injuries that were deemed severe enough by hunters to present to our emergency department. It is entirely conceivable that there were other nonfatal hunting injuries in the region during the period of study that never presented to our emergency department. Secondly, we only had access to cases of hunting injuries that were clearly labelled as such in the emergency department database. As mentioned previously, there is no national database of hunting accidents in Switzerland and therefore extrapolating our results to the rest of the country may not be entirely accurate. Finally, we cannot completely exclude the possibility that some cases of hunting-related injuries in the emergency department database were missed by the search criteria due to unstandardized data entry.

## 5. Conclusion

Nonfatal hunting accidents in Bern, Switzerland, are largely caused by firearms and falls and tend to occur in male hunters with a mean age of 50 years. The most common patterns of injury are orthopedic and head injuries, often requiring surgery. These findings are consistent with international studies of nonfatal hunting accidents but highlight a need for a national database documenting all hunting accidents in Switzerland.

## Figures and Tables

**Table 1:** Mechanisms of injury.

Mechanism of injury	Number	Percentage
Firearm projectile	4	21
Firearm backfiring	3	16
Knife	4	21
Fall	6	32
Other	2	10

**Table 2:** Patterns of injury.

Location	Number	Percentage
Head	7	37
Upper extremity	5	26
Lower extremity	5	26
Trunk	2	11
